# Feasibility and safety of laparoscopic resection for gastric GISTs larger than 5 cm: Results from a prospective study

**DOI:** 10.3892/ol.2015.3547

**Published:** 2015-07-30

**Authors:** FENG CAO, ANG LI, JIA LI, YU FANG, FEI LI

**Affiliations:** Department of General Surgery, Xuanwu Hospital, Capital Medical University, Beijing 100053, P.R. China

**Keywords:** laparoscopy, surgery, gastrointestinal stromal tumor

## Abstract

The role of laparoscopic resection for large gastric gastrointestinal stromal tumors (GISTs), particularly those >5 cm, remains under debate due the possibility of intraoperative tumor rupture. To determine the feasibility and safety of the laparoscopic approach in the treatment of large gastric GISTs, a prospective study was performed between March 2011 and March 2014. Intraoperative tumor rupture was studied as the primary outcome. Secondary outcomes were the conversion rate, surgical duration, estimated blood loss, time to tolerate fluid and solid diets, length of post-operative hospital stay and recurrence rate at the end of the follow-up. A total of 16 patients were included in this study, with a tumor size of 7.04±1.53 cm (range, 5.2–10.8 cm). No intraoperative tumor rupture occurred. The median duration of surgery was 88.1±31.9 min, with an estimated blood loss volume of 37.1±18.7 ml. No patient required a blood transfusion. The mean time until the start of oral intake for fluid and solid diets was 1.1±0.6 and 2.5±0.9 days, respectively. The median length of post-operative hospital stay was 5.4±5.8 days. The follow-up period for all patients was 16.9±11.2 months (range, 2–38 months). No local or distant recurrence was observed. The study indicates that laparoscopic resection for large gastric GISTs is feasible and safe. Laparoscopic surgery should be considered as the standard approach in all cases, irrespective of tumor size or location.

## Introduction

While a partial resection without lymph node dissection may be used as a curative treatment for gastric gastrointestinal stromal tumors (GISTs), laparoscopic surgery is a less invasive option when performed by experienced laparoscopic surgeons. In recent years, more and more studies have demonstrated that laparoscopic surgery is safe and effective in the treatment of gastric GISTs, irrespective of tumor size (1–7). Meta-analyses have also demonstrated that laparoscopic surgery for gastric GISTs is associated with a decreased blood loss volume, earlier return of bowel function, earlier resumption of diet, a shorter length of hospital stay and similar oncological results when compared with open surgery (8–10). However, laparoscopic resection for large gastric GISTs remains under debate in terms of the high risk of intraoperative tumor rupture. Guidelines from the European Society for Medical Oncology (ESMO) discouraged the use of a laparoscopic approach in with large tumors due to the risk of tumor rupture, which was associated with a high risk of relapse (11). The National Comprehensive Cancer Network (NCCN) guidelines considered laparoscopic surgery only when the GISTs were in favorable locations, such as the greater curvature or anterior wall of the stomach (12). In 2012, 38 GIST experts from the Chinese Society of Clinical Oncology discussed the treatment of GIST in Beijing and released an expert consensus (13). This consensus did not recommend a laparoscopic approach when the tumor size was >5 cm. Recently, retrospective studies from Asian countries demonstrated that laparoscopic surgery is feasible, safe and oncologically acceptable in the treatment of large gastric GISTs (14,15). However, evidence from a prospective study was lacking. The current study therefore presents the initial experience from a prospective trial.

## Patients and methods

### 

#### Patients

Following approval by the Ethics Committee of Xuanwu Hospital, Capital Medical University (Beijing, China), a prospective study was performed in the Department of General Surgery to evaluate the feasibility and safety of laparoscopic surgery in the treatment of 16 patients with large (>5 cm) primary gastric GISTs between March 2011 and March 2014. Written informed consent was obtained from each patient. Patients receiving any additional procedures at the time of the laparoscopic partial gastric resection that were not conducive to the recovery of bowel function, such as enterolysis, plus those with severe chronic medical issues (American Society of Anesthesiologists class IV or higher) (16) or any contraindications to laparoscopic surgery were excluded from the study. Continuous variables are presented as the mean ± standard deviation, however, statistical analysis of the data was not performed due to the small sample size.

#### Surgical procedure

Generally, a laparoscopic sleeve, wedge or distal gastric resection without regional lymph node dissection was applied to all the included patients. As described in previous studies (17,18), the location and growth pattern of the tumor, in addition to the tumor size, were the important factors in determining resectability through minimally invasive techniques. Exophytic masses located in the greater curvature, fundus and anterior wall of the stomach were easily amenable to partial resection, and one or two laparoscopic Endo-GIA™ (Medtronic, North Haven, CT, USA) staplers were used to remove the tumor. Exophytic tumors located in the posterior gastric wall were turned over towards the abdominal cavity after cutting the blood vessel around the greater curvature, then resected directly or by Endo-GIA stapler. Intraluminal tumors were directly resected using Endo-GIA staplers, with a wider extent of resection or an anterior gastrotomy to access and resect the tumor. Masses near the pylorus were removed by laparoscopic distal gastric resection. Intraoperative endoscopy was used to aid in tumor localization and avoid stricture of the cardia and pylorus. A closed suction drain placed around the surgical site was not used routinely. The specimen was retrieved in a plastic bag through a muscle-splitting incision in the left or right flank.

Post-operatively, nasogastric tubes were used routinely and removed at 12 h post-surgery. On the first post-operative day, the patients were encouraged to walk around the bed and drink water if there were no complaints associated with postoperative complications. Patients were discharged once they were able to tolerate a regular diet.

#### Primary and secondary outcomes

Intraoperative tumor rupture was studied as the primary outcome. Secondary outcomes were the conversion rate, surgical duration, estimated blood loss, time to tolerate fluid and solid diets, length of post-operative hospital stay and recurrence rate at the end of the follow-up.

#### Risk stratification following resection of the primary tumor

Risk stratification following resection of the primary GIST was performed according to the National Institutes of Health (NIH) risk stratification system (19).

#### Follow-up

According to the recommendations of the NCCN, patients who received a laparoscopic resection for GIST were followed up every 6 months by enhanced abdominal and pelvic computed tomography (CT) examinations for a 2-year period, then yearly thereafter (12).

## Results

### 

#### Patients

A total of 16 patients with a tumor diameter of >5 cm, underwent a laparoscopic resection for a gastric GIST in the Department of General Surgery between March 2011 and March 2014. The characteristics of these patients are shown in Table I. The mean age of the patients was 63.1 years (range, 46–71 years), and 10 of the patients were male. The most common signs or symptoms were anemia and gastrointestinal bleeding. All patients received abdominal and pelvic CT evaluation for primary and potential metastatic lesions. A total of 5 cases obtained a pre-operative definitive diagnosis of a GIST through endoscopic ultrasound-guided fine-needle biopsy, and 1 patient received pre-operative imatinib mesylate (400 mg/day) treatment for six months due to an excessive tumor size and multiple synchronous liver metastases.

#### Surgical procedure

**Figure 1.**
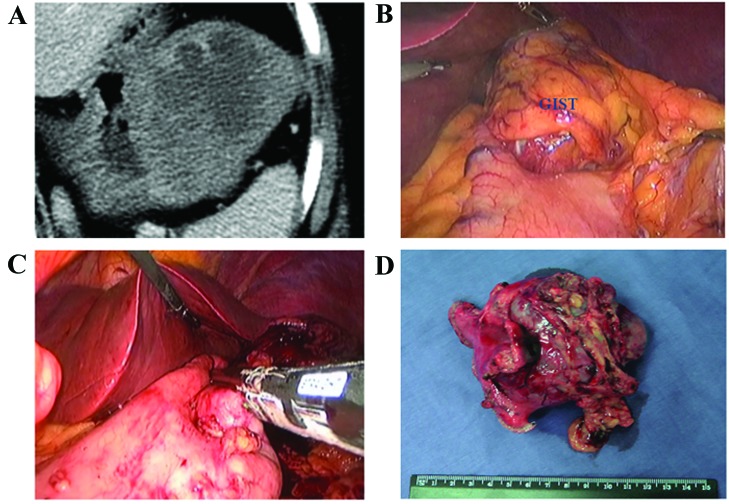
Laparoscopic sleeve resection for a GIST located in the gastric fundus. (A) Axial computed tomography showing an exogenous mass in the gastric fundus. (B and C) Intraoperative images. (D) Post-operative specimen. GIST, gastrointerstinal stromal tumor.

**Figure 2.**
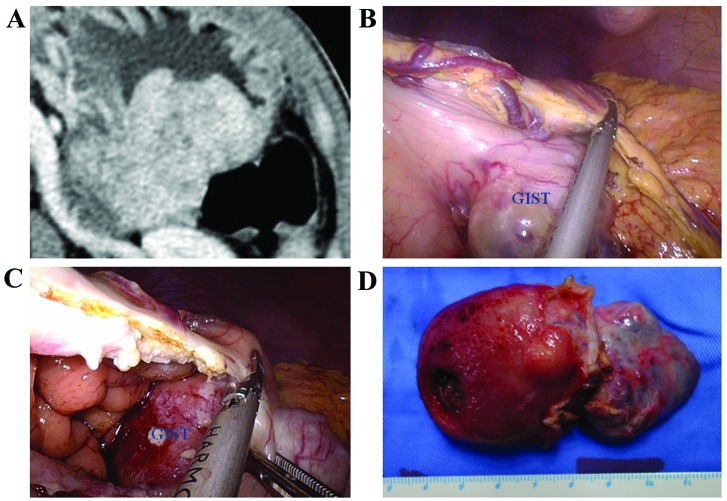
Laparoscopic resection for GIST located in the posterior wall of the stomach. (A) Axial computed tomography showing a heterogeneous mass in the posterior wall of the stomach with a mixed growth pattern. (B and C) Intraoperative images. After cutting the blood vessel around the greater curvature of the stomach, the tumor was turned over toward the abdominal cavity and resected directly using an ultrasonic scalpel. (D) Post-operative specimen. GIST, gastrointerstinal stromal tumor.

**Figure 3.**
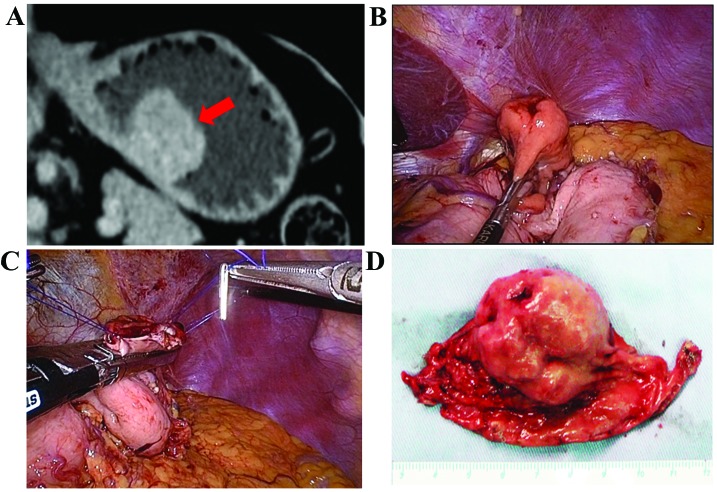
Laparoscopic resection for a gastric gastrointerstinal stromal tumor located in the posterior wall with an intraluminal growth pattern. (A) Computed tomography with coronal reconstruction showing an intraluminal mass located in the posterior wall of the stomach (arrow). (B and C) Intraoperative images. (D) Post-operative specimen.

All patients successfully underwent a laparoscopic gastric resection without conversion to an open procedure. In total, 9 tumors located in the greater curvature, fundus or anterior wall of the stomach with an exogenous growth pattern received laparoscopic sleeve or wedge resections (Fig. 1). Another 3 cases with exophytic tumors located in the posterior wall of the stomach were turned over toward the abdominal cavity and resected directly or by Endo-GIA (Fig. 2). Direct sleeve or wedge resections with a wider extent were applied to 2 patients with intraluminal tumors located in the anterior wall of the stomach body, and two tumors that were located in the posterior wall of the stomach with an intraluminal growth pattern were resected though an anterior gastrotomy (Fig. 3). The characteristics of the different surgical procedures are presented in Table II. When using the Endo-GIA resection of the tumors, 3 patients underwent an intraoperative endoscopy to avoid cardia stricture. A one-stage laparoscopic resection was performed in 1 patient for the gastric GIST and synchronous liver metastases. In total, 4 liver metastases were enucleated after 6 months pre-operative treatment. Incisional margin hemorrhage occurred in 4 patients and subsequent hemostasis was performed with clipping. Delayed gastric emptying occurred in 1 patient, which was resolved at post-operative day 27. All tumors were retrieved in a plastic bag though a muscle-splitting incision without rupture.

#### Primary and secondary outcomes

With regard to the primary outcome, no intraoperative tumor ruptures occurred. As for secondary outcomes, the median duration of surgery was 88.1±31.9 min, with an estimated blood loss volume of 37.1±18.7 ml. No patient required a blood transfusion. The mean time until the start of oral intake for fluid and solid diets was 1.1±0.6 and 2.5±0.9 days, respectively. The median length of post-operative hospital stay was 5.4±5.8 days (Table III).

#### Pathological finding and risk stratification following resection of the primary tumor

The mean tumor size for all patients was 7.04±1.53 cm (range, 5.2–10.8 cm). Positively staining for cluster of differentiation (CD)117, discovered on GIST-1, CD34 and S-100 was found in 15 (93.8%), 13 (81.3%), 10 (62.5%) and 2 (12.5%) patients, respectively. Since all gastric tumor sizes were >5 cm, risk stratification was only dependent on mitotic rate. According to the NIH risk stratification, 11 patients with tumors exhibiting a mitotic rate of <5/50 high-power fields were classified as the moderate-risk group. While the other 5 patients belonged to the high-risk group. All patients were administered imatinib (400 mg/day) therapy post-operatively for at least 1 year (Table IV).

#### Follow-up

The follow-up period for all patients ranged between 2 and 38 months (mean, 16.9±11.2 months). A total of 12 patients received one or more enhanced CT scan. No local or distant recurrence was observed in any of the patients and none of the patients succumbed to the disease.

## Discussion

The role of even single-port laparoscopic resection for relatively small gastric GISTs has been established by a series of retrospective cohort and comparative studies (1,2,6,7,17,20–24). In a study by Sexton *et al*, 61 patients received laparoscopic gastric resection for GISTs, with one conversion to an open procedure in order to control bleeding originating from the spleen. The mean tumor size was 3.8±1.8 cm and all but one case achieved an R0 resection. The mean surgical duration was 151.9±67.3 min, and the mean estimated blood loss volume was 97.4±200.7 ml (25). In a size-matched study (median size, 3.6 cm in laparoscopic group vs. 4.3 cm in open group), Karakousis *et al* found that the median length of stay post-surgery in the laparoscopic group was lower than that in the open group (4 vs. 7 days; P=0.002), as was estimated blood loss (25 vs. 100 ml; P=0.006). Median surgical duration, operative mortality, and 30-day morbidity and oncological outcomes were similar in the two groups (24). A long-term survival study also demonstrated favorable results for laparoscopic surgery, even in patients with large GISTs (2,26,27).

As aforementioned, the current guidelines or consensus from NCCN, ESMO and China discourage the use of laparoscopic surgery for large gastric GISTs (11,12). Guidelines from Japan also suggest that the laparoscopic resection of gastric GISTs should be reserved for patients with a tumor size of <5 cm and performed only by a skilled surgeon with complete familiarity with the neoplastic characteristics of gastric GISTs (28). The major concern with regard to laparoscopic surgery in the treatment of large gastric GISTs is the risk of tumor spillage or tumor capsule rupture, resulting in peritoneal seeding and a worse prognosis. However, De Vogelaere *et al* stated that the low morbidity rates and long-term disease-free period of 100% observed in their study cohort indicated laparoscopic resection to be a safe and effective method for treating gastric GISTs, irrespective of tumor size (5). Ronellenfitsch *et al* also demonstrated that the feasibility of laparoscopic wedge resection was not determined by tumor size, and that the indication for a laparoscopic wedge resection was not directly affected by the location of the gastric GIST (29). A size-matched study showed that laparoscopic resection for GISTs >5 cm was superior to an open procedure in terms of surgical duration, blood loss, time to ground activities, first flatus and liquid diet, and post-operative stay (15). In the Xuanwe Hospital, Capital Medical Hospital, laparoscopic surgery is the first choice of treatment for gastric GISTs regardless of the tumor size or location. In the present perspective study, laparoscopic resection for large gastric GISTs was found to be feasible and safe, without a requirement for conversion to open surgery. No intraoperative tumor rupture occurred and the application of the concept of fast-track surgery significantly accelerated patient recovery, with a post-operative hospital stay of 5.4±5.8 days. To avoid combined organ resection and major functional sequelae, guidelines recommend pre-operative imatinib treatment of differing durations dependent on the mutation type (11). In the present series, a 63-year-old female patient with a large, 15×10-cm, gastric GIST located near the gastroesophageal junction and synchronous liver metastases received a one-stage laparoscopic resection for the primary and metastatic tumors after 6 months of pre-operative therapy (30). This case was the first to demonstrate the feasibility of one-stage surgery for a giant gastric GIST and liver metastasis.

Although a laparoscopic approach for gastric GISTs has been considered as the gold-standard surgical treatment in recent series and reviews, laparoscopic resection for large gastric GISTs, particularly those >5 cm, remains a challenging technique. To avoid intraoperative tumor rupture, toothless forceps should be used to grasp the normal stomach wall. Valle *et al* demonstrated that large exophytic lesions can be protected from lacerations of the capsule by covering them with an endobag prior to starting the resection on disease-free tissue (5). A plastic bag should be used routinely when removing tumors from the abdominal cavity.

There are several limitations to the present study. Firstly, the characteristics of this cohort study meant that the short-term results of an open approach could not be obtained for comparison. Secondly, the limited number of cases and short duration of follow-up may have restricted the value of the conclusions. Thirdly, the tumors included in this study were mainly located within the fundus and greater curvature of the stomach, which were easy targets for laparoscopic surgery.

Irrespective of these limitations, the present results also suggested that laparoscopic resection for large gastric GISTs is feasible and safe. Laparoscopic surgery should be considered as the standard approach in all cases, irrespective of tumor size and location. Randomized controlled studies with long-term follow-up periods should be performed to confirm these results.

## Figures and Tables

**Table I.** Characteristics of patients with large gastric GIST tumors (>5 cm) who received laparoscopic resection.

Characteristic	Value
Mean age (range), years	63.1±4.2 (46–71)
Gender (male/female), n	10/6
BMI	24.5±2.1^a^
Presenting symptoms, n (%)	
Anemia	6 (37.5)
Gastrointestinal bleeding	4 (25.0)
Abdominal pain	3 (18.8)
Incidental finding	3 (18.8)
Preoperative GIST diagnosis, n(%)	5 (31.3)
Tumor size (range), cm	7.04±1.53 (5.2–10.8)
Tumor location, n (%)	
GEJ	2 (12.5)
Fundus	3 (18.8)
Great curve	6 (37.5)
Body	5 (31.3)
Growth pattern, n (%)	
Exogenous	12 (75.0)
Intraluminal	4 (25.0)

a Mean ± standard deviation. GIST, gastrointestinal tumor; BMI, body mass index; GEJ, gastroesophageal junction.

**Table II.** Characteristics of different laparoscopic approaches in treatment of large (>5 cm) gastric gastrointestinal stromal tumors.

Group	Tumor size, cm	Growth pattern	Surgical duration, min)	Estimated blood loss, ml	Time to tolerate solid diet, days	Length of post-operative hospital stay, days	Follow-up time, months
1 (n=9)	7.5±1.8	Exogenous	73.2±25.9	28.6±9.2	2.1±0.3	3.6±0.5	20.9±11.6
2 (n=3)	6.4±1.4	Exogenous	110.7±12.6	65.3±9.5	2.7±0.6	4.0±0.4	11.7±12.7
3 (n=2)	6.5±0.1	Intraluminal	69.6±0.7	17.5±10.6	2.0±0.0	15.0±16.9	18.0±4.2
4 (n=2)	6.3±0.7	Intraluminal	140.0±2.8	52.5±3.5	4.5±0.7	6.0±0.0	6.0±2.8

Group 1, laparoscopic sleeve or wedge resection; group 2, laparoscopic resection after turning over toward the abdominal cavity; group 3, laparoscopic sleeve or wedge resection with wider extent; and group 4, laparoscopic resection though anterior gastrotomy. Data are presented as the mean ± standard deviation.

**Table III.** Operative and post-operative characteristics.

Characteristic	Value
Intraoperative tumor rupture, n (%)	0 (0.0)
Conversion to open surgery, n (%)	0 (0.0)
Median surgical duration (range), min^a^	88.1±31.9 (45–142)
Estimated blood loss (range), ml^a^	37.1±18.7 (10–75)
Mean time to tolerate a fluid diet (range), days^a^	1.1±0.6 (0.5–3.0)
Mean time to tolerate a solid diet (range), days^a^	2.5±0.9 (2–5)
Median length of post-operative hospital stay (range), days^a^	5.4±5.8 (3–27)
Complications, n (%)	5 (31.3)
Anastamotic bleeding, n (%)	4 (25.0)
Delayed gastric emptying, n (%)	1 (6.3)

a Mean ± standard deviation.

**Table IV.** Pathological finding and risk stratification.

Parameter	n (%)
Cell type	
Spindle	11 (68.8)
Epithelioid	2 (12.5)
Mixed	3 (18.8)
Mitotic rate, /50 HPF	
≤5	11 (68.8)
5–10	4 (25.0)
≥10	1 (6.3)
Positive for	
CD117	15 (93.8)
DOG1	13 (81.3)
CD34	10 (62.5)
S-100	2 (12.5)
Ki-67 index, %	
<10	13 (81.3)
≥10	3 (18.8)
Risk stratification	
Moderate	11 (68.8)
High	5 (31.3)

CD, cluster of differentiation; DOG1, discovered on GIST-1; GIST, gastrointestinal stromal tumor; HPF, high-power fields.
